# Comparing competing characterizations suggests there might be more than one type of interest

**DOI:** 10.1038/s41598-024-70751-6

**Published:** 2024-08-28

**Authors:** Daniel Dukes, Catherine Audrin, Fabrice Clément, Marcello Mortillaro

**Affiliations:** 1 Swiss Center for Affective Sciences, University of Geneva, Campus Biotech, 9 Chemin des Mines, 1202 Geneva, Switzerland; 2 University of Teacher Education, Vaud, Switzerland; 3 University of Neuchâtel, Neuchâtel, Switzerland; 4 Swiss Distance University Institute, Brig, Switzerland

**Keywords:** Interest, Appraisal theory, Latent class analysis, Emotion, Psychology, Health care

## Abstract

Although there is general consensus concerning the importance and function of interest in our daily lives, there is little agreement about its nature. Four studies of increasing ecological validity (total N = 993) were carried out to compare two different characterizations of interest in terms of the key appraisals involved. The findings indicate that while a two-appraisal model is suitable to explain the interest we can feel towards simple stimuli, a more complex model may better capture the nature of interest in the real world. Further analysis suggested the contrasting previous results could be resolved by arguing that previous models of interest capture different types of interest. This novel finding represents a promising first step towards a more definitive definition of interest, and suggests that while interest may always be related to motivating exploration, learning and general well-being, researchers should be more precise about the type of interest to which they refer.

## Introduction

Many of us will have experienced finding a particular book, film, or piece of news particularly interesting, only to be surprised when close friends and colleagues dismiss it as boring or irrelevant. What is it, then, that makes something interesting?

Interest has been described as the ‘emotion associated with curiosity, exploration, intrinsic motivation, and information seeking’^1 p. 96^ and has been linked to successful learning both in infancy^2,3^ and adulthood^4^. Furthermore, maintaining an employee’s interest and motivation has been shown to be vital for the progress of any organization^5^, while a “markedly diminished interest or pleasure” is one of the main diagnostic criteria of Major Depression Disorder^6^.

Given the importance of interest in so many spheres of life, being able to characterize its true nature could be instrumental to finding out, for example, how to better motivate learners in education, improve working conditions for employees, and to inform researchers tasked with designing clinical interventions. Whereas in the past, educators, employers, and clinicians may have historically relied on intuition and experience to make things more interesting, a solid theoretical and empirically tested model of interest would surely enhance their efforts.

But despite interest’s undeniable importance for many walks of life, there is still some debate about its true nature^7,8^. For example, although there is general consensus among emotion researchers that interest qualifies as an emotion^9–15^, there are some important voices of dissent^16,17^. For example, two leading basic emotion theorists, failed to agree on the matter. While Paul Ekman described interest as “a cognitive state of focused attention”, rather than an emotion^16^, Carroll Izard saw “the emotions of interest and enjoyment as fundamental to the formation and maintenance of social ties”, among other attributes^12^. Meanwhile, the constructionist view on emotion focuses more on the role of language and culture in shaping emotions, and as such, the debate is somewhat different. Additionally, there is also the question about how an emotional interest compares to a more epistemic curiosity^18^, as some researchers have used these terms interchangeably^19^, while others have argued they are very different^20^. In any case, while certainly interesting, these differences of opinion are of little consequence here, as interest was unquestionably presented to participants as an emotion.

In this series of studies, we investigate what makes something truly interesting by employing a cognitive-appraisal model^21^, as doing so offers excellent explanatory power in terms of why we feel the way we do towards objects and events. This approach is underpinned by the assumption that “it is not the events per se that determine emotional responses, but evaluation and interpretations of events”^22, p. 162^. More generally, it is *as if* an emotional experience begins when the individual’s emotional processes answer a series of evaluative questions (see^23^). For example, the individual might evaluate, more or less consciously, how novel or unexpected an object or event is, whether it is something that is difficult to deal with, whether it is controversial or not, etc. The results of these evaluations determine the nature of the specific emotion that is experienced: the same set of appraisals always lead to the same emotions, while different appraisals generally lead to different emotions^24^.

Based on the results of a number of empirical studies, Silvia suggested that the nature of interest can be captured by as few as two different appraisals: a composite appraisal of ‘novelty/complexity’ and another of ‘coping potential’ (e.g.^25,26^). He argued that if an object is new and neither too difficult nor too easy to understand, then it is interesting^1,27^. However, a more recent examination suggests a more complex characterization^28^. As part of a much larger study assessing how 24 emotion words were understood in 27 countries in 24 different languages, interest was best captured by appraisals of ‘coping potential’, ‘goal relevance’ (how relevant the object/event is for personal goals) and ‘normative significance’ (whether the object/event conforms to conventions or typical standards). Specifically, characteristics of interest included positive coping potential, high goal relevance, and violation of norms^28, p.198^.

While these studies provide convergent evidence that an appraisal of ‘coping potential’ is a component of interest, there are obvious important differences in the results. The most likely explanation for these discrepancies is that in Silvia’s experiments, interest was principally related to visual triggers^8^, while Scherer and Fontaine^28^ investigated the overall semantic meaning of interest. In other words, the experimental design in each case may have led to context-specific conclusions.

Our goal is to better understand what underpins these conflicting characterizations of interest by investigating its appraisal structure across increasingly ecological contexts. This would be a first step in providing a comprehensive definition, for future clinicians, employers, and researchers to build on. We expect to find a characterization similar to the one suggested by Scherer & Fontaine^28^, with high novelty, high goal relevance, and norm violations.

In the first of a series of four studies, we will use a paradigm similar to that used in previous experimenter-led studies, using controlled stimuli in a laboratory setting. The second study will adopt a more ecological paradigm, with participants reporting their appraisals to real-world events they personally experienced. The third consists of a diary study, where participants freely report events and objects from their daily lives that they considered interesting, thus providing a broader range of subjectively valued, *interesting* objects and events. While each of these exploratory studies reached similar conclusions, a fourth, pre-registered study was carried out online using a validated questionnaire with many more participants to test these results. These studies were carried out in accordance with the relevant guidelines and regulations of the ethics committee of the University of Neuchâtel, Switzerland, which also approved the studies. Informed consent was received from all the participants before they began. The data collection was anonymous.

## Study 1: controlled stimuli

### Method

The study was conducted in the lab and was designed to ascertain the appraisal structure of interest in relation to reading.

### Participants.

Sixty-six participants (38 females, 28 males; mean age: 33.59 years, range: 18–76 years old) were recruited via Facebook through an announcement shared through the lab page. We expected a large effect size based on Silvia’s results (^27^ Cohen’s d = 0.78). According to Arend and Schäfer’s rules of thumb^29^, when there is a medium Intra Class Correlation (ICC), which is advised when there is no previous study providing any information regarding the ICC, effects can be detected from any combination between 50 participants with 3 items and 30 participants with 25 items. We measured 10 items for each participant and decided to recruit more than the suggested minimum.

### Material

Reading was chosen as the first focus of study because it is similar to the brief visual stimuli requiring cognitive evaluation used in Silvia’s previous experiments that led to the supposition of a two-appraisal structure of interest but nonetheless allowed for the possibility that other appraisals might be involved.

Participants were asked to read excerpts taken from *Men are from Mars, Women are from Venus*^30^, selected because it is generally interesting and readable according to online reviews, and it was unlikely that participants would have read it. Indeed, the passages proposed to the participants were the ten most frequently highlighted passages by e-book readers, highlighted presumably because the readers had thought they were interesting and/or informative. We acknowledge other explanations for the highlighting are possible, and a pre-validation of the study could have been beneficial to confirm our assumption. Given the nature of the stimuli (written passages), however, we can reasonably expect a strong impact of individual differences, and therefore, it would be very difficult to assume that pre-validation would give us sufficient or appropriate ground truth. Our primary concern was to ensure that participants found the passages interesting, and we can control that since we have a specific question about their interest level for each passage, which can be seen as a proxy of a “manipulation check”.

A survey was designed, and used across the first three studies, which instrumentalized the appraisals previously described in the literature as being explanatory of emotion events, namely, novelty, coping potential, intrinsic pleasantness, goal relevance and normative significance^31^. By “norm significance”, we mean whether the event is compatible with social and/or personal norms. The higher the score, the higher the incompatibility. Importantly, the choice of an original scale for our study came from the need to preserve the ecological validity of our studies. First, to be meaningful for the participants, our items needed to be context-specific, in a way that was not possible for a generic measure (a detailed presentation of the items used in the studies and a discussion of how the appraisals were operationalized and the rationale between these choices is described in the supplementary materials). Secondly, we aimed to capture the appraisal immediately after the event happened. This implies using a very brief and easily understandable measure—goals that most standardized questionnaires cannot achieve. We recognize that this may be seen as a limitation, which is why in Study 4, we used a standardized measure of appraisals.

The theoretically predicted appraisal sequence—corresponding to the order presented in the previous paragraph—was maintained when presenting the appraisals to the readers. While other sequences could have been experimentally feasible, we believe that appraisals should be presented following the logical sequence in which they are processed according to the theoretical model of reference to facilitate participants’ responses as done in other appraisal questionnaires [GAQ (the questionnaire can be retrieved at: https://www.unige.ch/cisa/files/3414/6658/8818/GAQ_English_0.pdf)]. Each appraisal was operationalized with one item in the first three studies, except for novelty in Study 3 (that covers a potentially wide spectrum of events). where an additional appraisal question was deemed necessary to capture both the ‘unfamiliarity’ and ‘unexpectedness’ components of that appraisal. One additional item related directly to how *interesting* the participant had found each event or stimulus (e.g., the chosen paragraphs of the book in Study 1) in order to analyze which appraisals correlated with the more global *interesting* scores. Responses were recorded on a seven-point Likert scale ranging from 1 (not at all) to 7 (very much). In Study 4, rather than using the origisurvey, which was adapted to fit each context, a validated questionnaire [the Geneva Appraisal Questionnaire (GAQ)] was used to evaluate emotional episodes more generally. For study 4 then, intrinsic pleasantness is measured by two items, novelty is measured with 3 items, and goal relevance is measured with two items. Normative significance is measured with 4 items. In this study, some items were reversed score for consistency in the meaning of the dimension across the studies. Finally, coping potential is measured with one item which referred to control. It is important to note that the order of the questions was not randomized, as this was specifically detailed in the manual of the GAQ. Copies of the original surveys (in French), including the items, appraisals and their translation can be found in the supplementary materials section (B–E) for each of the studies.

### Procedure

Upon arrival at the lab, the participants were given a description of the study and were asked to sign a consent form if they wished to continue. They were informed they could leave at any time without providing any justification. Once they had signaled their intention to participate, they were asked to read ten passages of the book (the order of which was randomized). After they had read each passage, they were asked to complete a survey before reading the next passage. There were, therefore, ten passages and ten surveys per participant. None of the participants reported having previously read the book.

### Results

The mean scores of interest and of the appraisals were calculated and are presented in Table 1 (below). We then compared two models to predict the ratings of interest: Model 1 used a two-appraisal model (similar, but not identical to the one suggested by Silvia) and Model 2 used a five-appraisal model (similar, but not identical to the one suggested by Fontaine, Scherer and Soriano).

**Table 1:** The mean scores (and standard deviations) of interest and the appraisal components for readers.

Emotion	Mean	SD	Appraisal	Mean	SD
Interest	4.759	1.72			
			Coping	5.276	1.518
			Novelty	3.778	1.516
			Intrinsic pleasantness	4.764	1.476
			Normative significance	2.698	1.616
			Goal relevance	4.676	1.514

The results presented in Table 2 indicate that when using a two-appraisal model (Model 1), both ‘novelty’ and ‘coping potential’ are significant predictors of interest. This supports the argument that both these appraisals are predictive of interest. However, a five-appraisal model (Model 2) appears to fit the data better as it explains more of the variance (Delta Chi^2^(3)  = 84.918, p < 0.001) and, interestingly, in a qualitatively different manner. Specifically, interest is better predicted by the larger model as a function of employing both a greater number of appraisals and a different variety of appraisals, since novelty, intrinsic pleasantness and goal relevance now predict interest, but coping potential no longer does.

**Table 2:** A comparison of the results between the two (Model 1) and five appraisal models (Model 2).

Fixed effects	Model 1					Model 2				
	b	SE	p-value	Lower 95% CI	Upper 95% CI	b	SE	p-value	Lower 95% CI	Upper 95% CI
Intercept	6.738	0.246	0.001	6.249	7.228	4.225	0.347	0.001	3.492	4.962
Coping	0.157	0.032	0.001***	0.094	0.221	0.047	0.033	0.152	−0.017	0.112
Novelty	−0.744	0.033	0.001***	−0.810	−0.676	−0.525	0.039	0.001***	−0.604	−0.446
Intrinsic pleasantness						0.250	0.04	0.001***	0.170	0.329
Norm significance						−0.033	0.037	0.277	−0.091	0.026
Goal relevance						0.248	0.037	0.001***	0.175	0.323
Random effects	σ2	SE				σ2	SE			
Participants										
Intercept	0.659	0.812				0.396	0.629			
Stimuli										
Intercept	0.016	0.127				0.001	0.014			

***- p<.001; **- p<.01; *- p<.05.

### Discussion #1

The results suggest that using a larger model may be more effective for characterizing interest, at least for this kind of interesting stimuli.

Contrary to expectations based on previous literature, ‘coping potential’ ceased to predict interest when additional appraisals were considered. This outcome may be a consequence of the material used in the study. For instance, it could be that more cognitively challenging stimuli, either more scientific books (i.e. less ‘pop’ psychology) or more abstract visual stimuli (e.g. Silvia’s stimuli in 2005^27^) for example, might have resulted in “coping-potential” also being a significant predictor of interest.

To further investigate these results, we conducted a more ecologically valid study in which both the setting and the stimuli varied in their cognitive requirements and were chosen by the participants themselves.

## Study 2: controlled events

### Method

This second study allowed further assessment of the hypothesis that the kind of material used as stimuli in the empirical study of interest may influence the results in terms of appraisal characterizations, particularly concerning the role of coping potential.

In study 2, we surveyed individuals either leaving a movie theater or an ice-hockey match.

### Participants

Power analysis was conducted using the pwr package^32^, and allowed us to determine the number of samples to detect a linear association at the 0.05 level, with 80% power. Results suggested that 79 participants were appropriate to detect a medium effect size. However, as we tested the impact of multiple predictors, we chose to collect more data (i.e., 200 participants) to attain similar power.

### Study 2a: movie

200 movie-goers (107 females, 93 males; mean age: 40.59 years, range: 18–84 years old) were surveyed as they left local movie theaters. Importantly, we tried to recruit any viewer exiting movie theaters; however, only those who agreed to participated are included in this sample. The questionnaire was really brief and all participants who started answering the questionnaire completed it. We do not claim that this sample is representative of all movie goers. For our research goal, the nature of the interesting event and sampling the emotional appraisals it elicits are more important than representing a specific population. The same rationale applies to study 2b.

### Study 2b: ice hockey

189 ice hockey spectators (75 females, 114 males; mean age: 32.48 years, range: 18–73 years old) were surveyed as they were leaving a hockey match at the end of one of three different matches.

### Materials

The participants were asked to complete a modified version of the survey described in the first study. The formulation of each item was modified when necessary to account for the differences in the nature of the events. In particular, for study 2a, the appraisal of coping potential was operationalized as complexity, as this is the key appraisal for Silvia when visual stimuli are presented ((as in “understanding”)).

### Procedure

The participants were surveyed by a research assistant as they either left a local movie theater or a hockey match. The procedure was explained, and upon agreeing to participate, individuals gave their consent. Participants were firstly asked to rate how interesting they found the particular event on a Likert scale from 1 ('not interesting at all') to 7 ('extremely interesting'). They then rated the event according to the appraisal dimensions.

### Results

The mean score of interest and of the appraisals was calculated and is presented in Table 3 (below). As in Study 1, regression analyses reveal that a five-appraisal model shows a significantly higher model fit than the two-appraisal model in explaining ratings of interest (Delta Chi^2^(3) = 27.219, p < 0.001 and Delta Chi^2^(3) = 73.557, p < 0.001 for movie and hockey studies, respectively), and that a different combination of appraisals predict interest.

**Table 3:** The mean scores (and standard deviations) of interest and the appraisal components for movie-goers and ice-hockey spectators.

Emotion appraisal dimension	Movie (N = 200)		Hockey (N = 189)	
	Mean	SD	Mean	SD
Interest	5.77	1.314	5.33	1.523
Coping	3.77	1.937	3.24	1.880
Novelty	4.11	1.675	4.80	1.551
Intrinsic pleasantness	5.37	1.354	5.09	1.490
Norm significance	3.57	1.612	3.97	1.622
Goal relevance	5.7	1.314	5.06	1.654

In the two-appraisal model (Model 1), while coping potential was a significant predictor of interest for both the movie and hockey conditions, novelty was not a significant predictor for movie-goers’ level of interest. Furthermore, the results (Table 4a, b) again show that using a five-appraisal model provides a better fit than the smaller model, and again, not only as a consequence of the fact there are more appraisals, but also in a qualitatively different manner. For instance, coping potential is not a significant predictor of interest for movie-goers, nor is novelty for hockey spectators.

**Table 4:** Regression coefficients for the reported interest and the appraisal components for movie-goers (4a) and ice-hockey spectators (4b).

	Model 1					Model 2				
	b	SE	p	Lower 95% CI	Upper 95% CI	b	SE	p	Lower 95% CI	Upper 95% CI
(a) Movies										
Intercept	4.849	0.289	0.001	4.278	5.420	1.875	0.413	0.001	1.059	2.690
Coping	0.199	0.046	0.001***	0.108	0.290	0.072	0.045	0.106	−0.016	0.161
Novelty	0.041	0.053	0.443	−0.064	0.146	0.009	0.045	0.845	−0.081	0.098
Intrinsic pleasantness						0.149	0.069	0.031*	0.014	0.285
Norm significance						0.180	0.053	0.001***	0.074	0.286
Goal relevance						0.374	0.073	0.001***	0.231	0.518

(b) Hockey										
Intercept	3.450	0.345	0.001	2.768	4.132	1.406	0.367	0.001	0.681	2.130
Coping	0.358	0.052	0.001***	0.255	0.461	0.137	0.037	0.001***	0.060	0.213
Novelty	0.150	0.063	0.018*	0.025	0.275	−0.026	0.045	0.565	−0.114	0.062
Intrinsic pleasantness						0.548	0.053	0.001***	0.443	0.653
Norm significance						−0.111	0.042	0.009**	−0.194	−0.027
Goal relevance						0.249	0.047	0.001***	0.156	0.342

***- p<.001; **- p<.01; *- p<.05.

### Discussion #2

In line with the first study, it appears that the appraisals which predict how interesting something is may vary according to the type of cognitive involvement. Based on our results concerning the role of coping potential, we can suggest that a Hollywood blockbuster may be amusing or entertaining without scoring very highly on interest at all; conversely, we can speculate that if we had surveyed people after watching a documentary, coping potential may have been necessary for it to be considered interesting. However, coping potential was a significant predictor of interest for ice-hockey spectators in both models. We can speculate that sports fans’ level of interest, including their feelings of coping, can be modulated by feeling that they can influence the outcome of the match by encouraging their team by shouting, chanting, or applauding louder. In this context, interest is directly related to subjective coping potential.

So far, our results suggest that the emotion of interest should be considered as more complex than has previously been proposed, and, that to some extent, what defines interest is context-specific. But even though Study 2 measured interest levels in the field, i.e., of people exiting a hockey match or a movie theatre, the choice of context was still limited. What interests people in real life is likely to be much more varied than previous studies have suggested, and, as a consequence, it may be that the nature of interest is more complex than has previously been proposed. Indeed, the hypothesis that that there may be different types of interest, would maps on to a recent paper about the diverse motives for human curiosity^33^.

To evaluate this possibility, we decided to inverse the rationale of the previous studies, by conducting a ‘bottom-up’ study in which we asked participants to report on events and occasions from their own lives in which they themselves experienced interest, rather than either having experiments impose an object of interest or chose a specific event.

## Study 3: diary study

### Method

This diary study involved participants completing a pre-prepared logbook concerning the objects that they found interesting in their daily lives.

### Participants

42 participants were recruited from social networks. As for study 1, we expected a large effect size based on Silvia’s results^27^. Based on Arend and Schäfer’s rules of thumb^26^, when there is a medium Intra Class Correlation (ICC), which is advised when there is no previous study providing any information regarding the ICC, effects can be detected from any combination between 50 participants with 3 items and 30 participants with 25 items. We measured 14 items for each participant in this study and decided to recruit around 40 participants. While 42 participants initially took part in the study, one logbook was incomplete and was thus discarded. The following analysis is thus based on the 41 remaining participants (30 females, 11 males; mean age: 30.78 years, range: 21–62 years old).

### Material

The participants were asked to complete a modified version of the survey described in the Study 1 and Study 2 for each of the events that they reported in the logbook.

### Procedure

Each participant was provided with a logbook that began with a full explanation of the task and a page for them to confirm their consent to continue or not. This was followed by some questions about the participant’s demographic details (age, gender, level of education). Details were then given about the precise nature of the procedure.

The participants were asked to briefly describe at least two things that they found interesting that day in 50 words or less, before completing a survey about that event. They were asked to do this for seven consecutive days. Participants were also encouraged to send photographs of the event if it could help their description. In fact, very few participants did this, and those that did, only did so occasionally. No further mention will be made of the photographs.

Participants were given very little indication of what could constitute an ‘interesting’ thing. The only direction they were given was as follows (in French): “What can be interesting? Objects, events, people, ideas… Everything can be interesting.” Wording it in this way was done to encourage use of a lay person’s view of what can be determined as interesting, rather than supplying stimuli for participants to evaluate. For each event reported, participants were asked to rate the appraisals of novelty (including unfamiliarity and unexpectedness), coping potential, normative significance, goal relevance and intrinsic pleasantness. The questionnaire used is reported in the supplementary materials.

### Results

The mean score of interest and of the appraisals was calculated and is presented in Table 5 (below). For this particular study, given the large variability that we expected int terms of events described, it was relevant to capture two separate components of novelty, ‘(un)familiarity’ and ‘(un)expectedness’. We thus operationalized novelty with two appraisal questions concerning familiarity and expectedness, reverse scored, and averaged the responses. As for study 1, the results reported in Table 6 indicate that when using a two-appraisal model (Model 1), both novelty and coping potential are significant predictors of interest. As in the previous studies, a five-appraisal model (Model 2) explained significantly more variance (Delta Chi^2^(3) = 219.77, p < 0.001) and did so in a qualitatively different manner than the model with only two appraisals. Consistent with the results for the movie-goers and in a very similar manner to the results for the hockey spectators, the appraisals of intrinsic pleasantness, normative significance and goal relevance were significant predictors of the level of reported interest.

**Table 5:** Appraisal scores for all events.

Emotion	Mean	SD	Appraisal dimension	Mean	SD
Interest	5.552	1.55			
			Coping	4.892	2.132
			Novelty	3.913	1.803
			Pleasantness	4.969	2.113
			Norm significance	1.646	1.462
			Goal relevance	4.728	1.998

**Table 6:** All diary events.

Fixed effects	Model 1					Model 2				
	b	SE	p-value	Lower 95% CI	Upper 95% CI	b	SE	p-value	Lower 95% CI	Upper 95% CI
Intercept	4.865	0.247	0.855	4.369	5.362	2.734	0.270	0.001	2.192	3.253
Coping	0.200	0.003	0.001***	0.142	0.258	–0.012	0.029	0.672	−0.070	0.045
Novelty	−0.069	0.034	0.042*	−0.137	−0.002	0.027	0.030	0.361	−0.031	0.086
Intrinsic pleasantness						0.414	0.029	0.001***	0.355	0.473
Norm significance						0.116	0.039	0.003**	0.038	0.194
Goal relevance						0.112	0.030	0.001***	0.053	0.172
Random effects	σ2	SE				σ2	SE			
Participants										
Intercept	0.395	0.628				0.218	0.468			
Stimuli										
Intercept	0.004	0.066								

***- p<.001; **- p<.01; *- p<.05.

In this study, we were further interested in assessing whether different types of interest could be highlighted based on their appraisals. The purpose of the following analysis was to class each event into groups of events according to the similarity of their appraisal structure. Thus, for this analysis we used event level data as previously performed by Patrick and colleagues^34^ and performed a latent class analysis on the five appraisals^35^. Rather than categorizing each event into a particular class (as in cluster analysis, for example), such analysis estimates the probability that such an event belongs to the particular class^36^. Furthermore, it can provide comparisons between different models (e.g., that provide a 3 or 4 category solution) and can determine which one characterizes the data better.

Models were estimated using the poLCA package^37^. We based our choice on statistical measures of fit and interpretability. More specifically, we compared models based on the Bayesian Information Criterion (BIC-^38^), where the lowest value indicates the best model fit. As we did not have a dichotomous indicator, we could not use the bootstrap likelihood ratio test to select the best model^34^. We further considered the Akaike information criterion (AIC ^39^). As for the BIC, the lowest value refers to the best model. We finally compared models based on their relative entropy. As explained elsewhere^40^, entropy represents the classification quality of each model, and the relative values of entropy range between 0 and 1, where the highest values indicate the clearest distinction among the classes.

As highlighted in Table 7 below, after analysis, the different indicators suggested different choices. While the BIC suggests the three latent classes model, the AIC favors the four classes model and the entropy the two classes model. Crucially, the models with 2, 3 and 4 classes all out-perform the 1 class model. This strongly suggests that there is more than one type of interest.

**Table 7:** Fit information for LCAs modeling with 2–4 latent classes.

Class(es)	Df	AIC	BIC	Likelihood ratio	Chi^2^	Entropy	LL
1	655	11,233.9	11,359.78	3938.955	42,236.58		−5586.95
2	624	10,742.87	11,019.16	2485.924	16,805.07	**0.800**	−5310.44
3	593	10,581.21	**10,997.92**	2262.269	15,010.79	0.774	−5198.61
4	562	**10,523**	11,080.12	2142.054	13,294.98	0.766	−5138.5

Unweighted n = 685.

*AIC* Akaike information criterion, *BIC* Bayesian information criterion, *LL* log-likelihood.

The lowest BIC is in bold.

The goal of this paper was to better understand what underpins conflicting characterizations of interest. This post-hoc analysis suggests that the answer might be that there is more than one type of interest. And, while we do not pretend to have uncovered enough evidence to suggest how many types of interest there might be, there is some evidence that that BIC is better at selecting the correct number of cases than AIC or entropy^38^, suggesting that the model with 3 classes is probably the most appropriate. We will use that model to tentatively illustrate what those three types of interest could be.

**Fig. 1:**
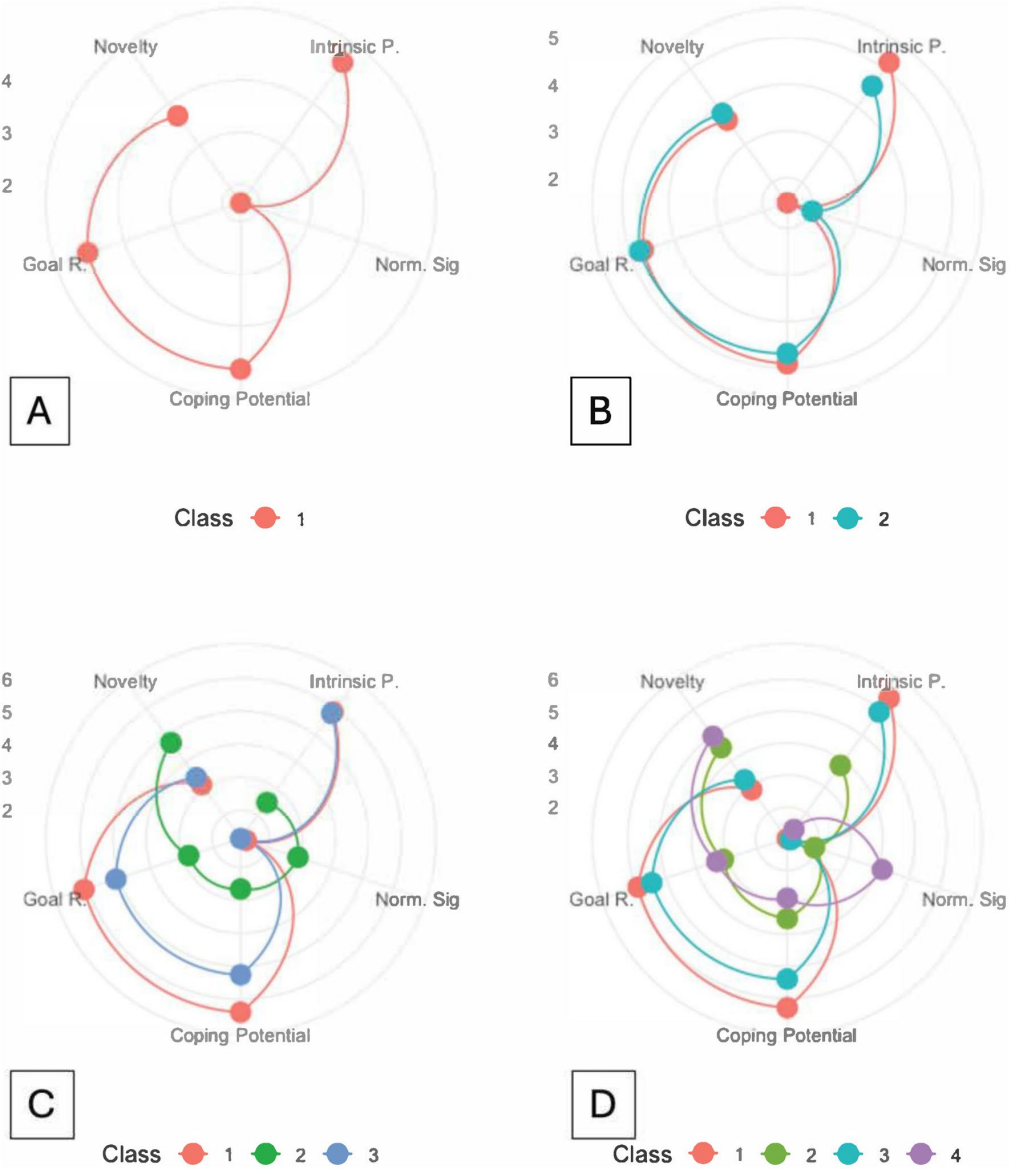
The four models, three of which (**B–D**) illustrate the classes drawn from the data suggesting different types of interest. Each of these three models was better than the model with only one class. In the three-class model (**C**), and as detailed in text, class 1 (in red) could be seen as *achievement interest*, class 2 (in green) as *morbid interest*, and class 3 (in blue) as *epistemic interest.* (**A**). *Goal R* goal relevance, *intrinsic P* intrinsic pleasantness, *Norm Sig* norm significance.

Figure 1 shows the results of the four class models featured in Table 7 in terms of the five appraisal scores. Focusing on the three-class model, the first type of interest (class 1), is characterized by high levels of intrinsic pleasantness, coping potential, and goal relevance but very low levels of norm incompatibility and novelty and, as such, could be characterized as a type of achievement interest, which implies enjoying facing a challenge (for example, imagine working on a task, or completing a sudoku). The second type of interest (class 2) has a relatively high level of novelty and norm incompatibility: a type of morbid interest, perhaps. In this we can include the typical interested attitude that people show when slowing down to observe a car accident on the highway more carefully. Finally, the last type of interest in our illustration (class 3) could perhaps be said to be a kind of epistemic interest, as it is characterized by high levels of intrinsic pleasantness, relatively high levels of coping potential, goal relevance, and novelty. Imagine in this case, the typical interest experience when reading a non-fiction book or watching a documentary.

Importantly, we recognize that this characterization of “types of interest” may not always be clear due to the many similarities between them, and interpreting differences is not straightforward. We suggest taking these descriptions as pointers for future studies specifically designed to uncover potential variations of interest, rather than as definitive conclusions. What is critical in addressing our research question is that the analysis confirmed the possibility of different appraisal structures when people report experiencing interest. This supports the argument that interest is a complex emotion, and that different types of interest may exist.

## Study 4: freely chosen events

The results of the three previous studies suggest two key findings. First, a five-appraisal model is better than a two-appraisal model for evaluating the emotion of interest, not only because it explains significantly more variance but because it does so in a qualitatively different manner. Second, the competing characterizations of interest may be explained by the existence of more than one type of interest. While the three studies used the same questionnaire based on context-specific items chosen for specific events (reading, going to a movie, watching an ice-hockey match, and events recorded in a diary), the objective here was to test the results of the three studies using a more comprehensive, validated measure of emotion and with a larger sample size. To do so, we collected (1) more data (sample size = 496 participants) and (2) used a theory-based widely-accepted measure of appraisal (GAQ). This study was pre-registered with the hypotheses based on the findings of the previous studies (https://osf.io/rkaeg).

### Method

This online study involved participants completing an online questionnaire regarding a specific event they had found interesting in their daily lives.

### Participants

518 participants were recruited on Prolific (https://www.prolific.com/). Among those, 496 answers were kept—as 22 participants either did not complete the study or answered a control question incorrectly. The final sample was then constituted of 243 females, mean age = 33.93, range = 18–74 years old. The sample size was pre-determined based on existing studies investigating the necessary sample size to perform latent profile analyses, which target a sample size of around 500, which should be enough to correctly identify the number of latent profiles^41–43^.

### Material

Participants were asked to think about an event that they had found interesting recently. They then completed the Geneva Appraisal Questionnaire^44^. This questionnaire is anchored in Scherer's Component Process Model of Emotion^44^ and allows assessment of an individual's appraisal process regarding a specific emotional episode. After completing the questionnaire, participants were asked to rate the intensity of the emotional episode on a scale ranging from 1 (not intense at all) to 5 (very intense). This represents the measure of interest.

### Procedure

Each participant was provided with a comprehensive explanation of the task with a consent page to confirm whether they wished to proceed. This was followed by a set of demographic questions (age, gender, level of education). Detailed information about the procedure was then provided. The participants were then asked to briefly describe in a few sentences an event that triggered their emotional before completing the survey about that event.

### Results

We first computed the appraisal scores for the five dimensions based on the guidelines of the Geneva Appraisal Questionnaire. Intrinsic pleasantness was measured by two items (e.g., “At the time you experienced the emotion did you think that the event was pleasant?”, omega = 0.87), novelty was assessed by 3 items (e.g., “At the time you experienced the emotion did you think that you could have predicted the occurrence of the event?”, omega = 0.58), goal relevance was assessed with two specific items (e.g., “At the time you experienced the emotion did you think that the event would have important consequences for you?”) while the other items more broadly addressed the goal significance dimension (omega = 0.59). Normative significance was measured with 4 items, (e.g., “At the time you experienced the emotion, did you think that the actions that produced the event were morally and ethically acceptable?”, omega = 0.75), while coping potential was measured with one item (i.e. “At the time you felt the emotion, did you think that the consequences, real or potential, of the event could be avoided or modified by appropriate human action?”), which referred to control. For this appraisal, we chose to keep only this item for reasons of coherence with the previous studies, as the other items pertained to different dimensions of control, namely power and adjustment. A full list of the items used for each appraisal can be found in the supplementary materials section.

As reported in Table 8 (below), results from multiple linear regressions of the two-appraisal model emphasize that novelty is a significant predictor of interest. As in the previous studies, a five-appraisal model (Model 2) explained significantly more variance (R^2^ = 0.18 versus R^2^ = 0.007) and in a qualitatively different manner than the model with only two appraisals.

**Table 8:** All recorded events (n = 496).

	Model 1			Lower 95% CI	Upper 95% CI	Model 2			Lower 95% CI	Upper 95% CI
	B	SE	P			b	SE	p		
Intercept	3.888	0.165	0.001	3.686	4.238	3.099	0.285	0.001	2.852	3.757
Coping	0.358	0.052	.398	−0.066	0.026	−0.033	0.02	0.151	−0.07	0.018
Novelty	0.150	0.063	0.023*	0.012	0.168	0.045	0.04	0.249	−0.024	0.130
Intrinsic pleasantness						0.023	0.03	0.718	−0.052	0.074
Norm significance						0.026	0.04	0.249	−0.154	0.025
Goal relevance						0.262	0.031	0.001***	0.209	0.330

***- p<.001; **- p<.01; *- p<.05.

In this study, we were interested in assessing whether different types of interest could be highlighted based on their appraisals. The purpose of the following analysis was to classify each event into groups according to the similarity of their appraisal structure. Thus, we performed Latent Profile Analysis (LPA) on the five appraisals^42^.

Data were continuous^45^ and estimated using the tidyLPA package^46^. As already indicated in Study 3, BIC is the most prominent indicator of fit and the lower the values the better the model^42^. To estimate the models, we also consider entropy indices, which describe how accurately the cases are classified in their true profile solutions (i.e., the highest value of entropy reflects the best distinction between the profiles).

**Fig. 2:**
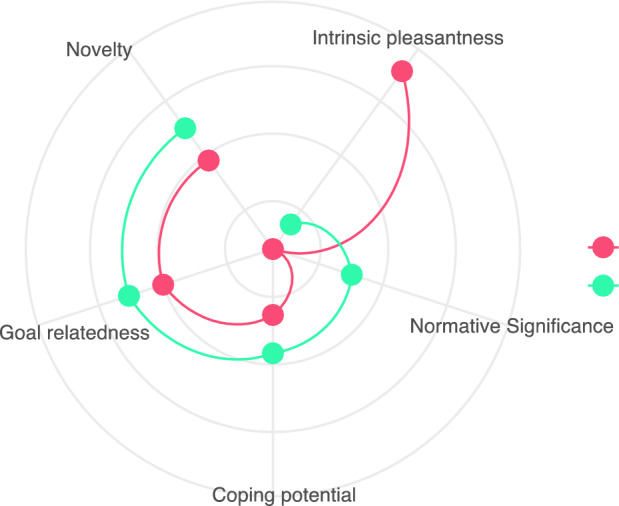
Graphic illustration of the two profiles of interest. Here, Profile 1 (in red) seems to be a combination of classes 1 (*achievement interest*) and 3 (*epistemic interest*) identified in Study 3, while Profile 2 (in blue) seems similar to class 2 (*morbid interest*) in Study 3.

Most indicators converge on a two-profile solution (Table 9), confirming previous findings that there is more than one type of interest. These results confirm our preregistered hypothesis that it is possible to identify more than one type of interest, which relate to different patterns of appraisal. https://osf.io/rkaeg. The two-profile solution (266 participants in class 1 and 91 participants in class 2) is depicted in Fig. 2. The first profile seems a combination of classes 1 and 3 identified in Study 3, while the second profile seems similar to what we called morbid interest when discussing the results of Study 3.

**Table 9:** Fit information for LPAs modeling with 1–5 latent classes.

Class(es)	AIC	BIC	Entropy	BLRT p-value
1	5838.93	5877.70	1.00	
2	5486.76	**5548.80**	**0.98**	.01
3	**5484.85**	5579.16	0.66	.01
4	5495.78	5604.36	0.71	.91

*BIC* Bayesian information criterion, *BLRT* bootstrapped likelihood ratio test.

Values in bold represent best-fit values.

## Discussion and conclusion

Given the importance of interest for effective learning and for general well-being, a well-defined model of what it means for something to be interesting could prove extremely informative. However, existing models conflict concerning the components that best predict interest. This set of studies compares two models of different complexity with a view to understanding what might explain the differences as a first step towards offering a better, more precise definition of interest.

One of the previous models proposed that the nature of interest could be captured using two appraisals^25,27^. This general finding was generally supported here, as, on the whole, both appraisals of novelty and coping potential were positive predictors of interest. However, when more appraisals were included, the predictive power of the two appraisals was greatly reduced, and an improved prediction of interest was achieved using different appraisals (see Table 8). This would suggest, as Silvia anticipated^27^, that more complex stimuli may reveal a more complex structure of interest than a two-appraisal model.

To explain this discrepancy, we argue that the contexts in which the stimuli are presented should be considered more carefully. Importantly, we do not mean to suggest that reading is necessarily linked to one type of interest and watching a movie another: a movie could be interesting, for example, aesthetically, or because it is informative, or because it challenges social norms. To paraphrase one of cognitive appraisal theory’s key assumptions, it is not the event that elicits a particular emotion but rather how we evaluate or interpret that event. In other words, it is not the movie itself that is interesting or not, but how we evaluate a particular aspect of that movie.

We should acknowledge that it was not always clear how to best instrumentalize each appraisal, such as capturing, for example, ‘coping potential’ when someone happens upon a new, interesting recipe, or to describe how one feels when finding a particular building interesting in terms of goal relevance or even norm significance. Of course, these labels can be perfectly justified in certain circumstances—perhaps the sauce is spicy, and you are unsure whether you can cope with it, or you are a scholar of architecture, studying the baroque church in a foreign city for an exam: in these particular cases, those appraisals make sense.

Consequently, it became apparent that it is important to let the participants choose their own stimuli and context when trying to characterize interest. In fact, post-hoc analysis of the final study confirms that there is more than one *type* of interest. We described one of these types found in the first three studies as ‘morbid interest’ as it was characterized by a high level of novelty and a comparatively high level of norm incompatibility. The existence of a morbid interest would be in line with a previous empirical study that shows that interest does not always need to be pleasant^47,48^ and with more recent work on morbid curiosity^49^.

The observation that multiple appraisal patterns can be linked to the same emotion, may seem to contradict the assumption of appraisal theory. However, we think this is not the case for at least three reasons: first, when considering the first three studies (Table 10), we can see that the appraisals that are crucial for the experience of interest are remarkably consistent in both the two-appraisal and the five-appraisal models, showing more commonalities than differences and confirming a rather stable appraisal structure for interest. Second, there could be minor variations within an appraisal pattern for the same emotion, possibly due to individual differences^23^. Third, there may simply be different emotions for which we use the same term. We expect future studies to try to address these possible explanations with a more tailored approach.

**Table 10:** Summary table of results from the two models of appraisal from the first three studies. Predictive appraisals for each survey are marked by an ‘x’.

Stimuli	Two appraisal model		Five appraisal model				
	Coping potential	Novelty	Coping potential	Novelty	Intrinsic pleasantness	Norm significance	Goal relevance
Reading	x	−x		−x	X		X
Movie	x				X	X	X
Hockey	x	X	x		X	−x	X
Diary study	x	X			X	X	X

Another question for future studies relates to the order of the questions. It may be that the order in which the questions were asked might affect the relationship between the appraisal dimensions and interest. While we think this is unlikely, it is possible that this has an impact and should, therefore, be tested.

We do not claim to offer a new definition of interest. We do not pretend either to have definitively uncovered two or three types of interest, as aiming to do this would require additional studies using different designs. However, we suggest that the assumption of a fixed directional relationship between specific appraisals and the experience of interest, which we also made at the beginning of our series of studies, may be incorrect. Our findings suggest that interest is more complex than previously acknowledged in the relevant literature and that there may indeed be different types of interest characterized by varying sets of appraisals whose influence on the emotional experience may substantially vary. This idea was operationalized in emotion recognition experiments by Tomkins and McCarter sixty years ago^50^ where interest was included in a ‘family’ with ‘excited’, ‘attentive’ and ‘alert’. We believe this is the first time that there appears to be empirical evidence for this claim. It is hoped that these findings will be considered a first step toward a consistent and adequate theory of interest, thereby informing educators, employers, clinicians, and perhaps even poets, about the true nature of interest.

### Supplementary Information

Supplementary Information.

## Data Availability

The datasets generated and analysed during the current study are available from the corresponding author on reasonable request.
