# Supplementary material for: Comparing competing characterizations suggests there might be more than one type of interest
Source: Sci Rep. 2024 Aug 28;14:19949. doi: 10.1038/s41598-024-70751-6 (PMC11358424; doi:10.1038/s41598-024-70751-6)
Supplement: Supplementary file 1 — Supplementary Information. [file 41598_2024_70751_MOESM1_ESM.docx]

SUPPLEMENTARY MATERIALS for

Comparing competing characterizations suggests there might be

more than one type of interest

Daniel Dukes^1*^, Catherine Audrin^2^, Fabrice Clément^3^ and Marcello Mortillaro^1,4^

1. Contextualized operationalization of appraisals
2. Original version of questionnaire and English translation for Study 1
3. Original version of questionnaire and English translation for Study 2a
4. Original version of questionnaire and English translation for Study 2b
5. Original version of questionnaire and English translation for Study 3
6. Cluster profiles of interest scores
7. Items of the GAQ used in Study 4

Translations from the original questionnaire have been added (in red). The appraisal titles have also been added to indicate which appraisal relates to which question (in blue). Neither appeared on the original questionnaires.

1. Contextualized operationalization of appraisals

One of the guiding principles of our research was to be as ecological as possible in the kind of events observed and how to collect data. This requirement matched with the fact that our research covers studies from very different contexts, made it necessary to develop contextualized items, at least for some dimensions: in other words, we couldn’t use generic items of, for example, novelty as this would be too undefined. Let’s consider the example of the hockey match: it would not be sensible to simply ask if the match was novel, but we had to find a more specific way in which novelty was applicable to this context and made sense to the people who were interviewed immediately after the match. In this case, the assumption was that the level of predictability during the match would be the best way to contextualize the appraisal of novelty. For the passages in the book, conversely, we could not ask about predictability (or whether this was new for the reader, which was a control variable) because we used only excerpts and the whole story of the book is not presented (in this latter case, predictability would have worked). So for study one, we operationalized novelty as originality, which is probably better for capturing the novelty appraisal in this context.

In particular, three appraisals were operationalized using different words, potentially referring to different sub-dimensions included in each appraisal. The words used in each study for these three appraisals are presented in Table S1 (below).

Please note that in study four, where contextualization is impossible, and we asked each participant about only one event, we used an extended theory-informed questionnaire of appraisal (GAQ), which uses multiple items for each appraisal, thus providing a much more complete, robust measure. More precisely, intrinsic pleasantness is measured by two items, novelty is measured with 3 items, goal relevance is measured with two items as they specifically related to relevance, whereas the other items referred more to the goal åsignificance dimension, Normative significance is measured with 4 items, while coping potential is measured with one item which referred to control. Further justifications and explanations are given in the text.

**Table S1**

A comparison of the words used in Study 1 (Book), Study 2 (Movie), Study 2b (Hockey Match) and Study 3 (Diary Study).

|  | Novelty #1 | Novelty #2 | Normative Significance | Goal relevance |
| --- | --- | --- | --- | --- |
| Book | Original |  | Shock | Goal obtention |
| Movie | Predictable* |  | Controversial | Goal obtention |
| Hockey match | Predictable* |  | Controversial | Identifiable |
| Diary Study | Familiar | Expected* | Controversial | Goal obtention |

**Novelty**

While it is possible to describe a book as ‘novel’ because it is original, the same could only be said of a hockey match in terms of how different it is from previous matches – when a hockey match is said to be “original,” we think this is better captured by the word “unpredictable” in everyday language. One less appealing solution would be to say that every live hockey match, seen for the first time, is original. But then, what would the measure of originality be? Presumably, it's predictability. While these words – novel, predictability, (un)familiarity and unexpectedness – are not exactly synonymous, we argue that they are very good proxies for novelty given how they need to be applied to different events and specific contexts. Indeed, in the GAQ (see study 4), each of these terms is used to more generally capture the meaning of novelty. Our choice of specific words was based on the context.

**Norm significance**

The same adjustment for the context (reading a book rather than seeing a live event) can explain our choice of wording for norm significance.

In study 1, considering the specificity of the written materials used, we found that the word “shocking” was better to describe violations of norms. We realized that this word was not appropriate for other contexts, so we did not use this operationalization in the other studies, where we preferred to use the word “controversial.”

Regarding reading a book, and considering the French linguistic context, shocking and controversial seem synonymous to us, one more focused on the reaction and the other on the quality of the material read. However, we do concede that it was probably unnecessary to make this distinction and that were we to run this experiment again, we could use “controversial” rather than “shocking” for the book reading task.

1. Original version of questionnaire and English translation for Study 1.

**Merci d’indiquer en encerclant de 1 (pas du tout) jusqu’à 7 (extrêmement), à quel point vous êtes d’accord avec ces phrases.**

**Please circle to indicate from 1 (not at all) to 7 (extremely) the point to which you agree with these sentences**

Novelty

*J’ai trouvé ce passage original*

*I found this passage to be original*

1 2 3 4 5 6 7

Intrinsic Pleasantness

*J’ai trouvé ce passage agréable à lire*

*I found this passage to be agreeable to read*

1 2 3 4 5 6 7

Coping (reverse-scored)

*J’ai trouvé ce passage facile à lire*

*I found this passage easy to read*

1 2 3 4 5 6 7

Norm significance

*Ce passage choquerait beaucoup de monde*

*This passage would shock lots of people*

1. 2 3 4 5 6 7

Goal relevance

Imaginez les raisons que quelqu’un pourrait avoir de lire ce livre.

A quel point vous estimez que leurs buts seront atteints ?

Imagine the reasons that someone could have to read this book.

To what extent do you say that their goals would have been obtained.

1. 2 3 4 5 6 7

Level of interest

*J’ai trouvé ce passage intéressant*

*I found this passage interesting*

1. 2 3 4 5 6 7
2. Original version of questionnaire and English translation for Study 2a

**Merci d’indiquer en encerclant de 1 (pas du tout) jusqu’à 7 (extrêmement), à quel point vous êtes d’accord avec ces phrases.**

Novelty (reverse scored)

*J’ai trouvé ce film prédictible*

*I found this film predictable*

1 2 3 4 5 6 7

Intrinsic Pleasantness

*J’ai trouvé ce film agréable à voir*

*I found this film agreeable to watch*

1 2 3 4 5 6 7

Coping Potential

*J’ai trouvé ce film complexe*

*I found this film complex*

1 2 3 4 5 6 7

Norm significance

*J’ai trouvé ce film controversé*

*I thought this film was controversial*

1 2 3 4 5 6 7

Goal relevance

*Pensez aux raisons pour lesquelles vous êtes venu voir ce film en particulier. Ce/Ces but(s) ont-ils été atteints?*

*Think about why you came to see this particular film. Were your goals obtained*

1 2 3 4 5 6 7

Level of Interest

*J’ai trouvé ce film intéressant*

*I found this film interesting*

1 2 3 4 5 6 7

1. Original version of questionnaire and English translation for Study 2b

**Merci d’indiquer en encerclant de 1 (pas du tout) jusqu’à 7 (extrêmement), à quel point vous êtes d’accord avec ces phrases.**

*Intrinsic pleasantness*

*J’ai trouvé ce match agréable à voir*

*I found this match agreeable to watch*

1 2 3 4 5 6 7

Novelty (reverse scored)

*J’ai trouvé que le déroulement du match était prédictible ou « cousu d’avance »*

*I found that the match progressed in a predictable fashion*

1 2 3 4 5 6 7

Norm significance

*J’ai trouvé la façon dont s’est déroulé le match controversé*

*I found the match progressed in a controversial way*

1 2 3 4 5 6 7

Coping potential

*J’ai le sentiment, comme supporter, d’avoir influencé le cours du match*

*I had the feeling, as a supporter, to have influenced the match*

1 2 3 4 5 6 7

Goal relevance

*Je me suis identifié à mon équipe*

*I identified with the team*

1 2 3 4 5 6 7

Level of Interest

*J’ai trouvé ce match intéressant*

*I found the match interesting*

1 2 3 4 5 6 7

1. Original version of questionnaire and English translation for Study 3

**Merci d’indiquer en encerclant de 1 (pas du tout) jusqu’à 7 (extrêmement), comment vous jugez ce passage.**

Level of Interest

L’objet est intéressant pour moi

The object is interesting for me

1 2 3 4 5 6 7

Novelty #1 (familiarity) – reverse scored

L’objet est familier pour moi

The object is familiar to me

1 2 3 4 5 6 7

Novelty #2 (unexpectedness) – reverse scored

L’objet est attendu

The object is unexpected

1 2 3 4 5 6 7

Intrinsic pleasantness

L’objet est agréable/plaisant pour moi

The object is agreeable/pleasant for me

1 2 3 4 5 6 7

Coping potential

J’avais contrôle de la situation et de mon environnement

I had control of the situation and of my environment

1 2 3 4 5 6 7

Norm significance

J’estime que l’objet est controversé

I reckon that the object is controversial

1 2 3 4 5 6 7

Goal relevance

Pensez aux buts que vous aviez au moment que vous avez vu l’objet.

A quel point vous estimez que vos buts sont atteints ?

Think about the goals that you had at the moment you saw the object.

To what extent would you say that your goals were obtained.

1 2 3 4 5 6 7

Please note that the score of novelty results from the mean of the reverse scores given to the items Novelty #1 and Novelty #2

1. Cluster profiles of interest scores (study 3)

| Appraisal\|Cluster | 1 | 2 | 3 | 4 |
| --- | --- | --- | --- | --- |
| Coping Potential | 0.420 | -0.715 | 0.484 | -1.121 |
| Novelty | 0.734 | 0.669 | -0.879 | 0.214 |
| Intrinsic pleasantness | 0.315 | -1.395 | 0.594 | -0.706 |
| Normative significance | -0.376 | 2.301 | -0.401 | -0.237 |
| Goal Relevance | 0.014 | -0.495 | 0.655 | -1.011 |

1. Items of the GAQ used in Study 4

| **Appraisal** | **Item**  (5-point scale from “not at all” to “extremely”) |
| --- | --- |
| **Intrinsic pleasantness** | How would you evaluate this type of event in general , independent of your specific needs and desires in the situation you reported above?  - pleasant |
|  | How would you evaluate this type of event in general, independent of your specific needs and desires in the situation you reported above? - unpleasant |
| **Novelty** | At the time of experiencing the emotion, did you think that the event happened very suddenly and abruptly? |
|  | At the time of experiencing the emotion, did you think that you could have predicted the occurrence of the event? |
|  | At the time of experiencing the emotion, did you think that you were familiar with this type of event? |
| **Goal Relevance** | At the time of experiencing the emotion, did you think that the event would have very important consequences for you? |
|  | At the time of experiencing the emotion, did you think that that it was urgent to act immediately? |
| **Normative significance** | At the time of experiencing the emotion, did you think that the actions that produced the event were morally and ethically ° acceptable? |
|  | At the time of experiencing the emotion, did you think that the actions that produced the event violated laws or social norms? |
|  | At the time of experiencing the emotion, was your behavior consistent with the image you have of yourself? |
|  | At the time of experiencing the emotion, did you think that real or potential consequences of the event were or would be unjust or unfair? |
| **Coping potential** | At the time of experiencing the emotion, did you think that real or potential consequences of the event could have been or could still be avoided or modified by ° appropriate human action? |
